# A Systematic Review of Telehealth Utilization for Bowel Management Programs in Pediatric Colorectal Surgery

**DOI:** 10.3390/children11070786

**Published:** 2024-06-27

**Authors:** Elizaveta Bokova, Ismael Elhalaby, Seth Saylors, Irene Isabel P. Lim, Rebecca M. Rentea

**Affiliations:** 1 Comprehensive Colorectal Center, Department of Surgery, Children’s Mercy Hospital, Kansas City, MO 64108, USA; 2 Tanta University Hospital, Faculty of Medicine, Tanta University, Tanta 31527, Egypt; 3 Department of Surgery, University of Missouri-Kansas City, Kansas City, MO 64108, USA

**Keywords:** telehealth, telemedicine, virtual, bowel management, Hirschsprung, anorectal malformation, constipation, incontinence, antegrade continence enema, cloaca

## Abstract

Recent advancements in pediatric surgery have embraced telehealth (TH) modalities, transitioning from traditional in-person consultations to virtual care. This shift has broadened access to healthcare, potentially enhancing affordability, patient and caregiver satisfaction, and clinical outcomes. In pediatric colorectal surgery, telehealth has been effectively utilized to support Bowel Management Programs (BMPs) for children suffering from constipation and fecal incontinence. A systematic review was conducted to assess the effectiveness of virtual BMPs, analyzing studies from January 2010 to December 2023, sourced from MEDLINE (via PubMed), Embase, and the Cochrane Library, with five studies included. Remote BMPs, implemented through video or telephone consultations, reported satisfaction rates exceeding 75% among families, indicating a strong preference for virtual interactions over traditional visits. Significant findings from the studies include improvements in Vancouver and Baylor scores, reductions in the duration of multidisciplinary consultations, enhancements in pediatric quality of life and Cleveland scores, and decreased frequency of laxative treatments. The implementation of TH has facilitated patient-led care, enabling timely adjustments in treatment and efficient distribution of medical supplies. The findings suggest that virtual BMPs are a viable and effective alternative to conventional approaches, yielding high caregiver satisfaction and superior clinical outcomes while promoting patient independence.

## 1. Introduction

Bowel Management Programs (BMPs) constitute specialized, week-long interventions tailored for pediatric patients with conditions such as Hirschsprung disease, anorectal malformations, and functional constipation [1,2,3,4,5]. The efficacy of BMPs is attributed to their customized approach, which includes systematic follow-ups, regular monitoring of stooling patterns, radiographic assessments to ensure adequate colonic emptying, and the ability to make timely modifications to the bowel regimen as necessary [4,6,7].

BMP implementation has historically faced significant barriers, including limited access to specialized healthcare providers and logistical challenges such as extensive travel and time commitments required for in-person consultations [8]. A recent study in Canada revealed that patients had to travel up to 120 km to access pediatric surgical care [9]. In low- and middle-income countries, patients endure prolonged journeys of over 12 h by public transport, followed by two days on foot to reach surgical care [10].

These challenges have prompted a pivotal shift towards telehealth (TH) services in pediatric surgery, transitioning away from traditional face-to-face interactions [1,11]. In the domain of pediatric colorectal surgery, telehealth has proven instrumental in facilitating the remote administration of BMPs. Telemedical technologies, including telephone, email, and real-time video conferencing, are being increasingly utilized for scheduling, counseling, and medication prescriptions [12]. This systematic review examines the role of telehealth in BMPs, underscoring its potential to improve clinical outcomes, enhance patient autonomy, and sustain high satisfaction levels among families.

## 2. Telehealth Applications in Pediatric Surgery

The recent health pandemic and subsequent lockdowns marked a significant inflection point in the adoption of telehealth within pediatric surgery [13,14,15,16]. For many healthcare providers, telemedicine utilization transitioned from a supplementary option to a critical necessity. Telehealth has proven particularly effective in remote settings, where it facilitates the provision of care to pediatric patients and significantly reduces costs associated with travel, time away from work, and childcare [17,18]. Additionally, virtual technology integration in surgical practices has provided numerous benefits to surgeons. These include more flexible scheduling options, improved clinical efficiency, and reduced travel demands, particularly for surgeons affiliated with multiple institutions [19,20,21].

### 2.1. Outcomes of Telehealth in Pediatric Surgery

Telehealth integration into remote and critical access hospitals has facilitated access to pediatric surgical expertise [14,15] and optimized transfer protocols from rural emergency departments. This method has been effective in enhancing patient care by decreasing the time required for provider evaluations and reducing unnecessary transfers between emergency departments and intensive care unit admissions [22].

**Figure 1:**
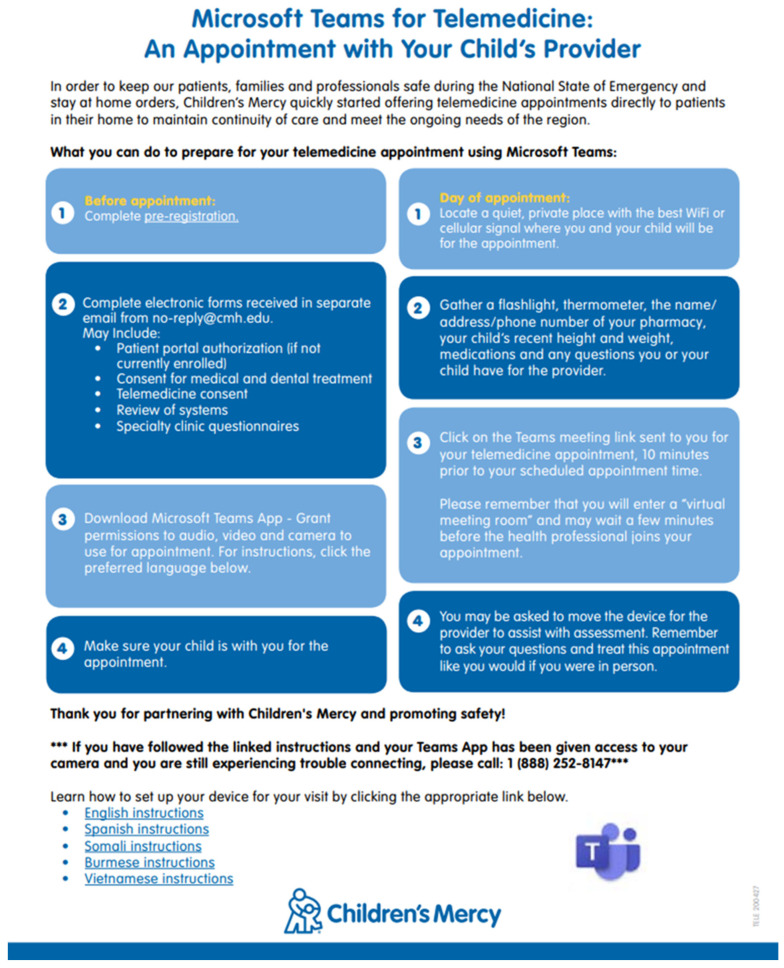
Stepwise instructions provided to the families prior to their telemedical bowel management appointment. Modified with permission from Lopez JJ et al. [23].

A comprehensive study analyzing telehealth outcomes in pediatric surgery, which included over 800 virtual consultations, demonstrated a significant reduction in wait times for urologic consultations via telehealth compared to in-person visits (7 min vs. 23 min) [20]. Additionally, for new patient consultations, the interval between the request for an appointment and the actual visit was substantially shorter with telehealth than with traditional face-to-face consultations (6–15 days vs. 30–180 days, respectively) [12]. To ensure timely and efficient healthcare delivery, providing families with precise instructions for telehealth visits is crucial. An illustrative set of such instructions from a representative institution is shown in Figure 1.

Another potential cost-effective benefit of telehealth utilization is the access to multidepartmental collaboration and education. Educating healthcare providers and sharing protocols during telehealth visits presents opportunities to expand their clinical skills, overcome geographic barriers to professional development [24], and potentially improve patient outcomes.

Families often face significant indirect costs when seeking pediatric surgical care, primarily attributed to hospital labor and travel requirements, lost wages, and childcare arrangements [25,26]. A systematic review underscored a significant increase in cost savings with TH compared to in-person visits (USD 345 vs. USD 49 per visitP) [26]. Overall, both caregivers and healthcare providers reported a high level of satisfaction with virtual visits, with a median satisfaction score reaching 10 (IQR 9-10) on a scale from 1 to 10 [20].

### 2.2. Challenges Associated with Telehealth in Pediatric Surgery

While telehealth offers numerous benefits, its implementation is not without challenges. Surveys indicate that some families experience discomfort using telemedicine during initial consultations, primarily due to concerns regarding the competence of physicians [18]. This apprehension often stems from a limited understanding of the standard practices involving surgeon collaboration and procuring second opinions. Despite technological advances, such sentiments underscore a persistent preference for in-person interactions with surgeons.

Additional impediments to the adoption of telehealth technologies in pediatric surgical practice include a range of technological, environmental, communicational, financial, and legal barriers. Moreover, the inherent limitations of telehealth, such as the inability to conduct physical examinations, pose significant challenges. These various barriers are comprehensively detailed in Table 1.

Remote healthcare encounters are fraught with several technological challenges that can compromise the effectiveness of telehealth. These include hardware malfunctions, unstable internet connections, and a lack of sufficient training for healthcare providers, state and locoregional licensing issues, all of which can impede effective communication [18]. Furthermore, multitasking and information overload during consultations may lead to misunderstandings and the partial loss of critical information. Financial constraints also play a significant role, as the costs associated with equipping healthcare facilities and homes with necessary telehealth technology can be substantial. Additionally, limited access to telehealth devices in certain regions compounds these challenges.

Privacy and legal compliance are critical concerns in telehealth, necessitating robust measures to protect personal health information and prevent data breaches [12,27,28,29,30]. The inability to perform comprehensive physical examinations during remote visits poses another significant barrier, especially for children with complex anatomical conditions [31,32]. Although sharing photographic images can mitigate some difficulties, in-person assessments remain crucial, particularly for conditions that may require surgical interventions [32,33].

To address these issues, telehealth for children with colorectal conditions is typically reserved for specific scenarios: following an initial in-person assessment, in the absence of postoperative anatomical concerns, and for cases involving mild malformation complexity [32,33]. Furthermore, implementing secure, HIPAA-compliant platforms, utilizing video demonstrations to assess anatomical concerns, and arranging in-person follow-ups for physical examinations have contributed to overcoming many of these obstacles. Federal improvements to reimbursement rates for telehealth services have further supported integration of these technologies into routine care [34].

## 3. Bowel Management: Understanding the Basics

Constipation and fecal incontinence present significant challenges in pediatric healthcare, adversely affecting quality of life, reducing independence, and increasing healthcare utilization [35]. Among patients with anorectal malformations (ARMs) or Hirschsprung disease (HD), the incidence of postoperative constipation is reported to range from 53 to 79%, with approximately 50% of these children experiencing fecal incontinence [7,36,37,38].

To address these issues systematically, the Bowel Management Program (BMP) was developed [1,8,9,10]. This week-long intervention, conducted by a multidisciplinary team, ensures comprehensive evaluation and treatment planning for the child, addressing associated concerns such as urinary continence and gastrointestinal care [39]. The BMP team typically includes pediatric surgeons, urologists, gynecologists, and gastroenterologists, along with physicians, nurses, advanced practice providers, coordinators, psychologists, and social workers, reflecting a broad spectrum of care [40,41].

Various protocols and scoring systems, such as the Baylor and Rintala Continence Scores for fecal continence and the Cleveland Score for constipation severity, are employed to evaluate BMP outcomes [42,43]. However, inconsistencies in reporting have historically impeded systematic outcome assessments. Recent trends indicate a shift towards standardized definitions and scoring systems, with diagnoses of constipation and fecal incontinence frequently based on Rome IV criteria [44]. Quality of life (QoL) assessments remain a pivotal aspect of patient-centered care, highlighting the emotional, behavioral, and social impacts of these conditions [1,42,45].

The effectiveness of BMPs in managing constipation and fecal incontinence is notable. Reports indicate that 87% of participants achieve stool continence by the end of the BMP week, with continence rates of 70–72% and 78% maintained at one- and two-year follow-ups, respectively [1,46]. Patients and caregivers have reported significant improvements in quality of life [47], evidenced by enhanced social adaptation, continuation of regular activities, and reduced emergency room visits and hospitalizations [1,47,48,49,50]. BMPs promote independence and community integration for the child [51], thereby enhancing their overall quality of life [5,52].

Despite these successes, the intensive nature of BMP care can be time-consuming and stressful for caregivers, potentially disrupting daily routines and lowering overall quality of life. This may lead to poor adherence to treatment protocols, resulting in increased hospital admissions and elevated morbidity and mortality rates [53,54]. Tailoring treatment to the child’s symptoms, preferences, priorities, available resources, and cultural context is crucial for successful outcomes [55].

## 4. Systematic Analysis of Telemedical Bowel Management Efficacy and Outcomes

### 4.1. Study Search

A comprehensive literature search covering the implementation of telemedical technologies in Bowel Management Programs (BMPs) from January 2010 to December 2023 was conducted across multiple databases, including MEDLINE (via PubMed), Embase, and Cochrane. This study did not require Institutional Review Board (IRB) approval due to its nature. Search terms included “Pediatric colorectal surgery”, “Hirschsprung disease”, “anorectal malformation”, “functional constipation”, “cloaca”, “constipation”, “fecal incontinence”, “bowel management”, “telemedicine”, “virtual”, and “telemedicine”, among other terms.

### 4.2. Study Selection

This systematic review adhered to the Preferred Reporting Items for Systematic Reviews and Meta-Analyses (PRISMA) guidelines for study selection. Additional relevant publications not identified in the initial search were located by examining the reference lists of the retrieved full-text articles. The inclusion criteria were articles that reported on the use of telemedicine (via video and/or telephone calls) within Bowel Management Programs (BMPs), with full-text versions available in English and involving pediatric, adolescent, and/or young adult populations (ages 0–21).

The exclusion criteria were as follows:

(1) Articles that exclusively addressed outcomes in adult populations, patients with colorectal cancer, or bowel management in children with inflammatory bowel disease (IBD).
(2) Review articles, book chapters, guidelines, preprints, and trial studies.
(3) Studies where virtual communication was used solely for survey data collection rather than routine clinical follow-ups.
(4) Manuscripts not available in full text in English.

## 5. Results

**Figure 2:**
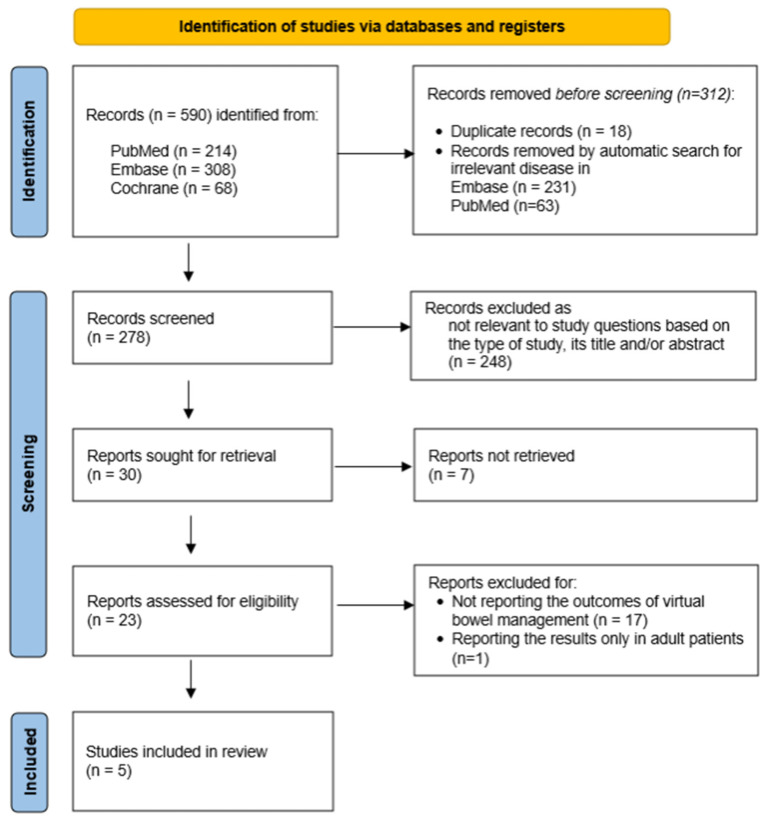
PRISMA flow diagram describing the study selection process.

An initial 590 articles were pulled from the literature search, with 312 studies removed before screening. Titles, abstracts, and study types were examined against the inclusion and exclusion criteria, with the full texts of potentially eligible studies obtained. Out of 30 abstracts meeting the inclusion criteria, 7 studies did not have a full-text manuscript available for analysis, and 18 were excluded for not having virtual BMP outcomes reported (n = 17) or including only adult patients (n = 1). Figure 2 illustrates the flow diagram depicting the article selection process. Article contents were subsequently separated and presented based on the aspect of TH BMP reported: applied telemedical BMP protocols and outcomes, including functional results, parental satisfaction rates, and resource utilization.

Five articles from the USA, the UK, Ireland, and Denmark were included in the systematic review. The study periods varied between June 2012 to June 2022. Most of the studies (n = 4, 80%) focused on TH use in follow-up BMP appointments [13,23,33,56], with one article covering specifically multidisciplinary visits [23]. One article described preclinic TH appointments performed by nurses to assess the patient prior to their further in-person clinic visit with a physician 4 weeks following the TH assessment [57].

In total, the studies included 439 children with colorectal conditions including HD (n = 76, 17%) [13,23,33], ARM (n = 141, 32%) [13,23,33], functional constipation, and spinal anomalies (n = 222, 51%) [13,23,56,57]. Two studies focused specifically on patients with FC [56] or bowel and bladder dysfunction (BBD) [57].

### 5.1. Protocols for Telemedical Bowel Management

Telemedical BMP visits were implemented in two formats: (1) completely remote BMP with no in-person appointments, and (2) hybrid BMP with an initial face-to-face clinic visit with further virtual follow-up consultations [13]. These BMPs were facilitated through video conferencing and telephone consultations [33,56,57] utilizing secure, virtual meeting software such as Microsoft Teams compliant with the Health Insurance Portability and Accountability Act (HIPAA) to safeguard protected health information (PHI) [56].

The multidisciplinary BMP teams comprised pediatric surgeons, pediatric gastroenterology and motility specialists, urologists, gynecologists, GI specialists, neurosurgeons, orthopedic surgeons, psychologists, nutritionists, and social workers. In addition to the physicians, advanced practice providers and nurse practitioners were involved in the child’s care. Secure data-protected software facilitated the inclusion of subspecialists during visits and enabled seamless communication among healthcare providers [13] to ensure a collaborative decision-making process.

Careful patient selection is essential to determine whether the child is eligible for remote bowel management. These criteria for telehealth BMP visits encompassed the following:

(1) Previously established care with the healthcare facility with an initial in-person visit [23,33,56].
(2) Prior delineation of anatomy through in-person evaluation, including examination under anesthesia and imaging studies [23,33,56].
(3) Absence of acute issues that may necessitate immediate admission to a healthcare facility [23].

Follow-up intervals post-BMP completion varied across studies, typically occurring at 1, 3, 6, and 12 months. Radiographic imaging and laboratory tests were conducted at local facilities [13,23], uploaded from a disc or submitted as a screenshot via e-mail or through the electronic medical record (EMR) patient portal, and interpreted by a hospital-affiliated radiologist prior to the virtual clinic appointment [13,23].

### 5.2. Outcomes of Virtual Bowel Management

#### 5.2.1. Bowel and Bladder Function

Functional outcomes following telemedical bowel management were reported in three studies [13,56,57]. These studies showed variability in the reporting criteria for fecal incontinence and the age range of the included patients, which posed challenges in standardizing outcome assessments. Notably, one study included patients as young as three years old, a factor that could impact continence assessments, as the typical age for readiness for toilet training is approximately four years. The lack of details regarding the toilet training status of these participants adds complexity to the interpretation of outcomes [13]. The other two studies assessed fecal and/or urinary continence in patients as young as two years old [56,57].

Statistical analyses demonstrated improvements in Vancouver and Baylor Scores at one and three months post-BMP completion. However, there were no significant changes in Cleveland and PedsQL scores or in the frequency of laxative treatments at the three-month follow-up [13]. One study noted that there was no correlation between quality of life (QoL) and the severity of malformations. The use of antegrade colonic enemas (ACEs) or the presence of an ostomy were the only factors negatively affecting QoL [33].

One year post-BMP, 69% of patients with functional constipation reported improved symptoms, with 79% meeting Rome III criteria for continence [56]. Urinary symptoms improved in 69% of the patients [57], and 74% achieved urinary continence by the three-month mark [13].

#### 5.2.2. Satisfaction Rates and Resource Utilization

Overall, telemedical BMPs were associated with high parental satisfaction rates, achieving up to 97% satisfaction [13,23]. This approach notably relieved stress for both children and their caregivers, with reported reductions in stress reaching up to 91% compared to in-person consultations. Most families reported that the quality of care delivered via telemedicine was comparable to, or better than, that received during in-person consultations. Furthermore, 75% of caregivers preferred telehealth over face-to-face BMPs.

However, there were reported challenges, including difficulties in obtaining and submitting radiographic imaging and a perceived loss of face-to-face social interactions with healthcare providers and other families [23]. Additionally, parents of children with severe malformations reported higher levels of anxiety, underscoring the need for targeted support during telemedical visits [30].

The implementation of telemedical BMPs also led to more efficient use of healthcare resources, significantly reducing the duration of multidisciplinary consultations compared to in-person visits—25 min versus 45 min, respectively—without impacting patient attendance rates [23].

## 6. Discussion

This systematic review highlights the substantial benefits and some challenges of implementing telemedical BMPs for pediatric patients with colorectal conditions. Our analysis reveals that telehealth can significantly enhance the accessibility and efficiency of bowel management, delivering care that is comparable to, if not exceeding, traditional in-person consultations in terms of patient and caregiver satisfaction [13,23]. Importantly, most caregivers favored telehealth platforms over conventional methods, underscoring the potential of telehealth to promote patient autonomy and reduce the logistic burdens associated with clinical care.

However, the interpretation of these findings must be considered alongside those of similar studies, which have also noted variability in the effectiveness of telemedical interventions depending on patient age and specific medical conditions [13,56,57]. Some studies have reported challenges related to the younger age of patients, which can affect the reliability of continence assessments and complicate outcome interpretations [13]. Moreover, while telehealth has been effective in reducing the duration of consultations and potentially lowering healthcare costs, issues such as difficulties in obtaining and submitting necessary diagnostic imaging and maintaining quality interpersonal interactions have been noted [23].

Strengths of the reviewed studies include the comprehensive nature of the telemedical BMPs, which encompass a variety of subspecialties and support services, facilitating a multidisciplinary approach to care [3,50,51]. However, the studies also exhibit limitations such as small sample sizes, lack of long-term follow-up data, and variability in (1) study designs and objectives, (2) healthcare systems among the four countries where the studies originated, and (3) outcomes assessment. The inclusion of patients with various diagnoses may impede the generalizability of the results. Additionally, outcomes were assessed using different scaling systems and telehealth technologies (e.g., phone vs. video calls). Some studies included patients as young as 2 years old, which can lead to measurement bias as these children had not reached toilet-training age at the time of continence assessment. Furthermore, the studies often did not account for variability in technological literacy among participants, which can influence the effectiveness of telehealth interventions.

Future research should focus on refining telehealth protocols to ensure consistent and reliable diagnostic assessments, particularly for younger children. Implementing standardized training programs for caregivers and healthcare providers can improve technological literacy and optimize the use of telehealth resources. Moreover, expanding the scope of telehealth studies to include diverse populations and longer follow-up periods will provide a more comprehensive understanding of its long-term benefits and limitations. Finally, addressing the emotional and psychological needs of families, especially those with severely affected children, should be integrated into telehealth strategies to enhance overall care effectiveness [33].

## 7. Conclusions

The implementation of telemedical bowel management serves as a viable alternative to traditional in-person BMPs for children experiencing constipation and/or fecal incontinence. This approach is associated with high satisfaction levels among caregivers and enhances patient autonomy, contributing significantly to improved patient care outcomes. Telemedicine offers increased convenience and accessibility, and ensures continuity of care, effectively addressing many of the challenges posed by traditional in-person consultations.

For telemedical BMPs to be successful, critical elements must be established, including a structured BMP protocol, secure software utilization, standardized outcome measurements, and the enhancement of technological literacy among all stakeholders. These components are essential for optimizing the effectiveness and efficiency of telehealth services in pediatric bowel management.

## Figures and Tables

**Table 1:** Barriers to telehealth in pediatric surgery. TH—telehealth.

Barriers to TH	Examples
Technological	Hardware malfunction
	Poor internet connection
	Digital literacy issues
2. Environmental	Distractions and interruptions
3. Communicational	Building provider-patient communication
	Communication etiquette
	Miscommunication via email or texts
	Coordination issues
	Information misinterpretation due to multitasking
4. Financial	Limited access to devices
	TH reimbursement issues
	Need for specific software
5. Legal	State-specific differences in TH regulations
	Necessity for the provider to be licensed in each state they practice in
	Privacy concerns
6. Lack of physical examination	

## Data Availability

No new data were created or analyzed in this study. Data sharing is not applicable to this article.
